# 40 Years of CSF Toxicity Studies in ALS: What Have We Learnt About ALS Pathophysiology?

**DOI:** 10.3389/fnmol.2021.647895

**Published:** 2021-03-18

**Authors:** Koy Chong Ng Kee Kwong, Pratap K. Harbham, Bhuvaneish T. Selvaraj, Jenna M. Gregory, Suvankar Pal, Giles E. Hardingham, Siddharthan Chandran, Arpan R. Mehta

**Affiliations:** ^1^Centre for Clinical Brain Sciences, University of Edinburgh, Edinburgh, United Kingdom; ^2^West Midlands Academic Foundation Programme, University of Birmingham, Birmingham, United Kingdom; ^3^UK Dementia Research Institute at University of Edinburgh, Edinburgh, United Kingdom; ^4^Euan MacDonald Centre for MND Research, University of Edinburgh, Edinburgh, United Kingdom; ^5^MRC Edinburgh Brain Bank, Academic Department of Neuropathology, University of Edinburgh, Edinburgh, United Kingdom; ^6^Edinburgh Pathology, University of Edinburgh, Edinburgh, United Kingdom; ^7^Anne Rowling Regenerative Neurology Clinic, University of Edinburgh, Edinburgh, United Kingdom; ^8^Centre for Discovery Brain Sciences, University of Edinburgh, Edinburgh, United Kingdom; ^9^Centre for Brain Development and Repair, InStem, Bengaluru, India; ^10^Nuffield Department of Clinical Neurosciences, University of Oxford, Oxford, United Kingdom

**Keywords:** amyotrophic lateral sclerosis, cerebrospinal fluid, motor neuron disease, neurodegeneration, pathophysiology, toxicity

## Abstract

Based on early evidence of *in vitro* neurotoxicity following exposure to serum derived from patients with amyotrophic lateral sclerosis (ALS), several studies have attempted to explore whether cerebrospinal fluid (CSF) obtained from people with ALS could possess similar properties. Although initial findings proved inconclusive, it is now increasingly recognized that ALS-CSF may exert toxicity both *in vitro* and *in vivo*. Nevertheless, the mechanism underlying CSF-induced neurodegeneration remains unclear. This review aims to summarize the 40-year long history of CSF toxicity studies in ALS, while discussing the various mechanisms that have been proposed, including glutamate excitotoxicity, proteotoxicity and oxidative stress. Furthermore, we consider the potential implications of a toxic CSF circulatory system in the pathophysiology of ALS, and also assess its significance in the context of current ALS research.

## Introduction

Amyotrophic lateral sclerosis (ALS), also known as motor neuron disease (MND), is a neurodegenerative condition that is pathologically characterized by the accumulation of ubiquitinated intracellular inclusions containing TAR DNA-binding protein 43 (TDP-43) (Neumann et al., 2006). Clinically, it initially manifests as focal weakness, rapidly progressing to widespread paralysis, with the resulting impairment in respiratory function usually being fatal. About 90% of ALS cases are believed to be sporadic, while monogenetic causes account for the remaining occurrences (Brown and Al-Chalabi, 2017). To date, various mechanisms such as glutamate excitotoxicity, impaired proteostasis and dysregulation of RNA metabolism have been implicated in ALS pathophysiology, with neurodegeneration believed to arise from their highly complex interplay (Hardiman et al., 2017).

## Early Suggestions of a Circulating Toxin

Although the pathophysiology of ALS was comparatively little understood in the 1970s, it had nevertheless already been recognized as a fatal neurodegenerative disease involving the selective loss of motor neurons (Wolfgram and Myers, 1973). Amongst various proposed explanations for the etiology of ALS was the existence of a circulating toxin—one thought to be particularly hostile toward motor neurons. Based on this premise, *in vitro* experiments were conducted, in which anterior horn cells were exposed to diluted serum from ALS patients (Wolfgram and Myers, 1973). Two important observations emerged from this study. First, ALS serum was considerably more toxic to anterior horn cells compared to serum obtained from patients with other neurodegenerative diseases. Second, the observed toxicity did not appear to extend to non-neuronal cells. The inability of the toxic factor to undergo dialysis prompted the authors to suggest the possibility of it being a protein, a particularly interesting proposal given that ALS was at the time not known to be a proteinopathy. The authors further noted a lack of association between patient demographics and serum toxicity.

Given the potential promise of these results in explaining the pathogenesis of ALS, subsequent experiments employing a range of culture systems, including motor neurons and neuroblastoma cells, were performed in the 1970s, in an attempt to reproduce previous findings (Horwich et al., 1974; Liveson et al., 1975; Lehrich and Couture, 1978; Viktorov and Bunina, 1979). These, however, failed to demonstrate evidence of serum toxicity in ALS, and it was only almost a decade later that findings of ALS serum toxicity were successfully replicated (Roisen et al., 1982). Intriguingly, the study investigators also observed increased neurotoxicity following exposure to serum from the relatives of ALS patients as well as from a number of neurological controls, suggesting that serum toxicity was not restricted to the ALS phenotype. In addition to confirming the specific toxicity of ALS serum toward anterior horn cells, the authors further concluded that the toxic factor was both non-dialysable and heat-labile. Other studies have also investigated the effect of ALS serum on various enzymatic reactions, with mixed results (Touzeau and Kato, 1983, 1986; Doherty et al., 1986; Maher et al., 1987; Van der Neut et al., 1991).

## From Serum to Cerebrospinal Fluid

Cerebrospinal fluid (CSF), which is often considered as an ultrafiltrate of plasma, is a clear colorless liquid that surrounds most of the central nervous system (CNS). In addition to providing buoyant support and protection to the brain, it also serves a regulatory role, made possible by substantial exchange of material between CSF and interstitial fluid (ISF). In view of the close proximity of CSF to neuronal cells, it was hypothesized that the toxin could also be present in the CSF circulation. The first study to assess the toxicity of ALS-CSF thus emerged in 1981 (Askanas et al., 1981). Measuring the levels of neuron-specific enolase (NSE), a marker of living neurons, in neuronal cultures treated with CSF obtained from ALS patients, however, revealed no significant toxicity. Two subsequent studies were also unable to demonstrate strong evidence of cytotoxicity following exposure to ALS-CSF, although a qualitative decrease in neuronal survival was observed in one study when exposure time was increased (Silani et al., 1987; Swift et al., 1988). Initial evidence of ALS-CSF toxicity arose only in 1993, with the observed toxicity found to be dependent on CSF concentration (Couratier et al., 1993). While additional studies were performed in the same decade (Iwasaki et al., 1995; Gredal et al., 1996; Terro et al., 1996; Smith et al., 1998), it could be argued that evidence for ALS-CSF toxicity remained mostly mixed, similar to serum toxicity.

**FIGURE 1:**
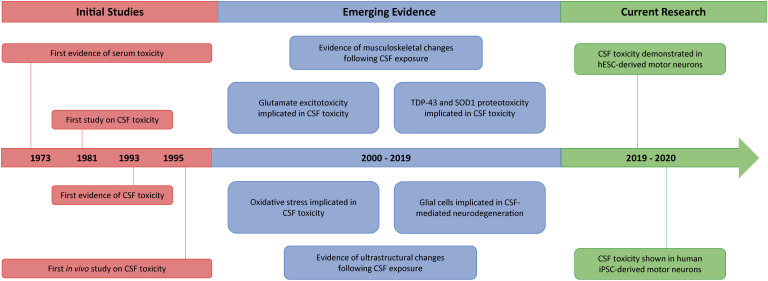
Timeline summarizing the key events from 40 years of CSF toxicity research in ALS. Following evidence of serum toxicity in ALS in the early 1970s, several studies started to explore the potential toxicity of ALS-CSF. By 1995, findings of CSF toxicity had been reported both *in vitro* and *in vivo*, although results were initially inconclusive. Subsequently, however, different mechanisms such as glutamate excitotoxicity, proteotoxicity and oxidative stress became implicated in CSF-mediated neurodegeneration, with a glial cell contribution also being demonstrated. Concurrently documented were various downstream pathological features, including ultrastructural, as well as musculoskeletal, changes. More recently, the toxicity of ALS-CSF has been found to extend to both hESC-derived and human iPSC-derived motor neurons.

From 2000 onward, however, evidence grew strongly in favor of the neurotoxic properties of ALS-CSF (see Table 2 in Ng Kee Kwong et al., 2020a). This was despite heterogenous study conditions, including different culture models, CSF concentrations and exposure times (see Table 1 in Ng Kee Kwong et al., 2020a). Notable was the finding that CSF from patients with both sporadic and familial forms of ALS caused cytotoxicity when incubated with neurons in culture. Although these results were predominantly based on rat neuron cultures, as well as the NSC-34 cell line (a mouse spinal cord-neuroblastoma hybrid cell line), recent evidence involving motor neurons derived from human induced pluripotent stem cells (iPSCs) and human embryonic stem cells (hESCs), indicates that CSF-mediated toxicity may also be common to human neurons (Sumitha et al., 2019; Brauer et al., 2020). Complementing *in vitro* evidence, several *in vivo* studies have also been performed, demonstrating a range of pathological changes upon CSF injection. In the following sections, we elaborate on the various *in vitro* and *in vivo* observations, and discuss the potential underlying mechanisms (Figure 1).

## Evidence of Excitotoxic Mechanisms

Glutamate excitotoxicity has been subject to longstanding interest in ALS research, and several studies have attempted to assess its contribution to CSF toxicity. Early suggestions of the possible involvement of excitotoxic mechanisms include the finding that 6-cyano-7-nitroquinoxaline-2,3-dione (CNQX), an antagonist of the alpha-amino-3-hydroxy-5-methyl-4-isoxazole propionic acid (AMPA)/kainite receptor, could protect against CSF-induced neurodegeneration (Couratier et al., 1993). Although the same study failed to demonstrate neuroprotection *via* blocking of *N-*methyl-D-aspartate (NMDA) receptors (Couratier et al., 1993), results from other groups suggest that both NMDA receptors and AMPA/kainate receptors may be implicated in CSF-induced neurodegeneration (Tikka et al., 2002; Sen et al., 2005). It was further shown that CSF-mediated neurodegeneration was preceded by an increase in intracellular calcium levels, with the rise being more prominent in motor neurons compared to other spinal neurons (Sen et al., 2005). Thus, glutamate receptor activation appears to be much more pronounced in neurons treated with ALS-CSF, with the variation across studies possibly arising from differences in glutamate receptor composition amongst employed neuronal cultures.

Concomitant experiments assessing glutamate levels in ALS-CSF as well as glutamate receptor activation following exposure to ALS-CSF have produced mixed evidence, with suggestions that elevated glutamate levels may be a feature of only a subgroup of ALS patients (Spreux-Varoquaux et al., 2002; Fiszman et al., 2010). Whether significantly raised glutamate levels are necessary to promote excitotoxicity is also unclear. In one study, the approximately threefold increase in glutamate levels observed in ALS patients was found to be sufficient to induce apoptosis and reduce viability in CSF-exposed cells (Cid et al., 2003). In another study, although ALS-CSF contained higher glutamate levels, and could promote neurotoxicity *via* ionotropic glutamate receptors, increased glutamate concentrations did not correlate with the degree of induced neurotoxicity (Fiszman et al., 2010). Conflicting findings were also reported by a different group, in which ALS-CSF was still shown to trigger excitotoxicity, as suggested by the protective effect of AMPA/kainate receptor antagonists, despite glutamate levels not being significantly elevated (Tikka et al., 2002).

Although it appears that glutamate excitotoxicity may form part of the overall neurodegenerative pathway, suggestions that elevated glutamate levels could be a driver of CSF-mediated neurodegeneration are not supported by other lines of evidence. First, neurodegeneration secondary to ALS-CSF exposure could not be reproduced by administration of L-glutamate in *in vitro* experiments (Anneser et al., 2006). Second, more recent results show that glutamate levels considerably higher than those found in ALS-CSF are required to produce neurotoxicity (Yanez et al., 2011). It is also worth noting that early findings demonstrating a possible neuroprotective effect by riluzole (Couratier et al., 1994), which remains the only globally licensed drug for ALS, could not be replicated (Yanez et al., 2011). Instead, the observed neurotoxicity could be inhibited by resveratrol, a natural antioxidant found in grapes, possibly by suppressing elevation of cytosolic calcium levels (Yanez et al., 2011). Other compounds found to reduce ALS-CSF toxicity include memantine, minocycline and lithium (Yanez et al., 2014). Intriguingly, the influence of all four neuroprotective factors appeared to be counteracted by the presence of riluzole. While the mechanistic underpinnings of these findings remain unclear, with riluzole potentially exerting both neuroprotective and neurotoxic influences in ALS, the authors suggest a possible confounding effect from riluzole in ALS clinical trials (Yanez et al., 2014). Lastly, we note that dialysis experiments, from which additional insights into the contribution of glutamate to CSF toxicity could potentially arise from, remain sparsely evidenced.

## Glial Contributions to CSF-Mediated Neurodegeneration

In line with the increasingly acknowledged role of astrocytes and microglia in ALS pathophysiology (Philips and Rothstein, 2014; Zhao et al., 2019; Clarke and Patani, 2020; Franklin et al., 2020), CSF toxicity has also been argued to possess a cell non-autonomous component. Suggestive findings arose as early as 1987 when *in vitro* experiments showed that ALS-CSF exposure triggered an increase in GFAP-positive cells, consistent with astrocyte proliferation (Silani et al., 1987). The cause of astrogliosis is unclear, although further results showed that it could follow from stimulation of mGluRs in response to ALS-CSF exposure, as suggested by the anti-proliferative effect of the mGluR group I antagonist, 1-aminoindan-1,5-dicarboxylic acid (AIDA) (Anneser et al., 2004). Complementing *in vitro* observations, ALS-CSF has also been found to promote increased GFAP immunoreactivity when injected in rats (Shahani et al., 1998). This time, the resulting astrogliosis was shown to be reduced by administration of the immunosuppressive agent, cyclophosphamide, or the selective monoamine oxidase B (MAO-B) inhibitor, (-)-deprenyl (Shahani et al., 2001, 2004). Nevertheless, given that a proportion of astrocytes may only become GFAP-positive following insult (Sofroniew and Vinters, 2010), increases in GFAP-positive cells could potentially be independent of astrocyte proliferation.

As the possible involvement of astrocytes further prompted researchers to investigate their role in glutamate excitotoxicity, one of the emergent findings was downregulation of the expression of glutamate transporter-1 (GLT-1/SLC1A2/EAAT2), which is expressed by astrocytes and is involved in glutamate uptake, following exposure to ALS-CSF (Shobha et al., 2007). Various mechanisms, such as abnormal mRNA processing and lipid peroxidation, have been proposed for the decreased GLT-1 expression. Expression of glutamate aspartate transporter (GLAST/SLC1A3/EAAT1) was, however, not found to be affected. More recent evidence showed that the reduction in glutamate uptake was also accompanied by a significant increase in the release of glutamate (Mishra et al., 2016). Nevertheless, whether the raised extracellular glutamate levels resulting from reduced glutamate uptake could contribute to neuronal death is still unclear.

Early findings have also implicated microglia in CSF-mediated neurodegeneration. Upon administration of minocycline, which blocks microglial activation, a neuroprotective effect was observed following CSF exposure (Tikka et al., 2002). Given that antagonists of NMDA receptors and AMPA/kainate receptors could also suppress the observed neurotoxicity, but without influencing microglial activation, the authors proposed that ALS-CSF may contain toxic factors that promote microglial activation, which, in turn, leads to the activation of ionotropic glutamate receptors and neuronal death. Nevertheless, the variable proportion of astrocytes and microglia across studies, which could range from five percent to thirty percent glial constitution, makes their exact contribution to CSF-induced neurodegeneration difficult to establish (Couratier et al., 1993; Tikka et al., 2002). In one study where microglia made up less than one percent of the total cell count for instance, neurodegeneration *via* a glutamate-dependent pathway could still be observed (Anneser et al., 2006).

The pro-inflammatory properties of ALS-CSF are further highlighted by its ability to upregulate in astrocytes the expression of various inflammatory factors, including interleukin 6 (IL-6) and tumor necrosis factor alpha (TNF-α), while increasing production of nitric oxide (NO) and reactive oxygen species (ROS) (Mishra et al., 2016). Expression of anti-inflammatory cytokines such as interleukin 10 (IL-10) was also found to be downregulated. The possible contribution of these changes to a neurotoxic environment is supported by the finding that conditioned medium from CSF-exposed astrocytes could promote neurodegeneration (Mishra et al., 2016). Similar observations have been made regarding microglia, with changes in the expression levels of both pro-inflammatory and anti-inflammatory cytokines, along with increased toxicity of microglia-conditioned medium toward neurons (Mishra et al., 2017).

Although many potentially toxic factors have emerged from studying the effects of ALS-CSF on glial cells, chitotriosidase-1 (CHIT-1), an inflammatory product specific to microglia, has been proposed to play a particularly important role in CSF-mediated neurodegeneration (Varghese et al., 2020). Proteomic analysis found CHIT-1 to be significantly upregulated in ALS-CSF compared to control CSF, suggesting its possible role as a biomarker (Varghese et al., 2013). This was subsequently substantiated by findings from the same group demonstrating its high specificity and sensitivity as a diagnostic biomarker (Varghese et al., 2020). It is worth commenting that the function of CHIT-1 in the human nervous system has still not been established, with CHIT-1 also being implicated in other neurodegenerative diseases, such as Alzheimer’s disease and multiple sclerosis (Varghese et al., 2020). Notwithstanding this, the particularly elevated levels of CHIT-1 in ALS-CSF, which have been found to promote astrogliosis and microgliosis, as well as neuronal loss, support its possible participation in CSF-mediated neurodegeneration (Varghese et al., 2020).

There are also intriguing observations derived from co-culture studies that suggest that the glial influence may be more complex than a direct contribution to neuronal loss. Although exposure of motor neuron mono-cultures to both ALS-CSF and control-CSF resulted in neuronal death, motor neurons co-cultured with glia experienced no significant decrease in survival following ALS-CSF exposure, with administration of control-CSF even leading to an increase in cell count (Barber et al., 2011). As well as emphasizing the role of glia in CSF-mediated neurodegeneration, these findings, as the authors proposed, suggest the presence of factors that may have a neuroprotective effect, in addition to toxic components in CSF.

## ALS as a Proteinopathy

While ALS is now recognized as a proteinopathy, proteotoxicity initially received comparatively little attention as a possible underlying mechanism in both ALS pathophysiology and CSF-mediated neurodegeneration. In fact, it was found that CSF toxicity could neither be suppressed by prior heating nor by using a 5 kDa filter, thus appearing to exclude the possibility of the toxic agent being a protein (Anneser et al., 2006). Much more recent evidence, however, supports the involvement of key ALS proteins, including superoxide dismutase 1 (SOD1) and TDP-43. Although CSF concentrations of SOD1 in ALS patients are not known to be significantly different from other individuals (Zetterstrom et al., 2011; Winer et al., 2013), misfolded SOD1 present in CSF obtained from patients with sporadic ALS has been shown to be highly toxic to NSC-34 cells, with antibody-mediated depletion to remove the misfolded SOD1 found to produce a significant reduction in neurotoxicity (Tokuda et al., 2019). Whether misfolding of the SOD1 protein is a phenomenon occurring inside cells or within the CSF circulation, as well as the factors promoting its occurrence, remain, however, incompletely understood.

With TDP-43 known to be present in ALS-CSF (Majumder et al., 2018), several attempts have also been made to establish the impact of ALS-CSF exposure on TDP-43 aggregation, and the associated downstream features. Intraventricular injection of CSF from ALS patients into rats resulted in the formation of cytosolic inclusions of TDP-43 co-localizing with ubiquitin (Gomez-Pinedo et al., 2018). In a later study, ALS-CSF injection was also found to induce TDP-43 proteinopathy as well as motor and cognitive disability in hTDP43 mice (Mishra et al., 2020). TDP-43 proteinopathy was, however, not observed in normal mice, although mild motor deficits were described. The authors further noted increased vulnerability with age, with 8-month-old mice experiencing a greater degree of muscle impairment when compared to younger mice. Earlier *in vitro* findings of TDP-43 mislocalization were again observed following ALS-CSF exposure, a feature which has previously been shown to be partially reversed by vascular endothelial growth factor (VEGF) supplementation (Shantanu et al., 2017). Other intriguing findings to emerge from the same study were the mislocalization of fused in sarcoma/translocated in liposarcoma (FUS/TLS) and formation of stress granules secondary to ALS-CSF exposure, with reversibility by VEGF further being demonstrated (Shantanu et al., 2017).

A more recent study employing human iPSC-derived motor neurons, however, failed to observe such changes following ALS-CSF exposure (Brauer et al., 2020). Additionally, although CSF exposure produced significant neuronal loss, this effect did not appear to be specific to cells treated with ALS-CSF. Nevertheless, exposure to ALS-CSF resulted in significantly higher Golgi fragmentation rates, possibly suggestive of an early sign of neurodegeneration. It has therefore been suggested that a dose-dependent effect may underlie the induction of pathological changes in cells (Brauer et al., 2020; Mishra et al., 2020). In support of this is the finding that TDP-43 mislocalisation and aggregation could be observed only when human glioma U251 cells were exposed to CSF from patients with both ALS and frontotemporal dementia (FTD), while CSF from ALS and control patients failed to induce similar pathology (Ding et al., 2015), consistent with the greater TDP-43 pathology seen in ALS-FTD.

Although additional evidence is required to establish the contribution of TDP-43 and SOD1 to the neurotoxicity of ALS-CSF, these findings could have important implications in our understanding of ALS pathophysiology. Of note is the possibility already suggested by others that the CSF circulation could constitute a pathway for the spread of pathology, which various lines of evidence are now hinting at (Smith et al., 2015; Gomez-Pinedo et al., 2018; Mishra et al., 2020). This could in turn help to potentially account for many of the unexplained features of ALS, one of which is the non-contiguous spreading pattern occasionally observed (Smith et al., 2015; Ng Kee Kwong et al., 2020b).

## Other Potential Candidates

Beyond the different factors already described, other components of ALS-CSF could also be responsible for its toxicity toward cells. Levels of 4-hydroxynonenal (HNE), a marker of lipid peroxidation, when raised to that of ALS-CSF, have been found to induce neuronal loss *in vitro* (Smith et al., 1998). Potentially elevated concentrations of 3-nitrotyrosine, as seen in patients with sporadic ALS, could also indicate oxidative stress by peroxynitrite, which has been shown to activate astrocytes and promote neurodegeneration (Tohgi et al., 1999; Cassina et al., 2002). While the contribution of oxidative stress as an underlying mechanism for CSF-mediated neurodegeneration remains unclear, with definite conclusions from studies measuring levels of oxidative stress biomarkers in ALS-CSF not being possible, antioxidants such as allopurinol and vitamin E have been found to suppress *in vitro* neurotoxicity following exposure to ALS-CSF (Terro et al., 1996). In addition to the various inflammatory cytokines with potentially neurotoxic properties, such as IL-6 and TNF-α (Moreau et al., 2005), immunoglobulin G (IgG) from ALS patients could be yet another contributing factor to CSF toxicity, given their previously evidenced role in promoting neurodegeneration and microgliosis (Obal et al., 2001; Pullen et al., 2004; Demestre et al., 2005). Finally, the possibility that accumulation of other metabolic waste products could result in a neurotoxic extracellular environment and CSF constitution cannot be excluded.

## Additional Downstream Effects of ALS-CSF Exposure

Several pathological changes have been observed across both *in vitro* and *in vivo* models in response to ALS-CSF administration. During the 1990s, increased neurofilament phosphorylation was reported when chick spinal cord neurons were exposed to ALS-CSF (Nagaraja et al., 1994), a finding subsequently reproduced *in vivo*, with the change being considerably more pronounced in ventral horn motor neurons (Rao et al., 1995). Despite being repeatedly linked to ALS-CSF exposure, the cause of neurofilament phosphorylation, as well as its contribution to CSF-mediated neurodegeneration, is still poorly understood (Vijayalakshmi et al., 2009). Ultrastructural changes following exposure to ALS-CSF also include Golgi fragmentation and endoplasmic reticulum stress, the latter being supported by free polyribosomes and fragmented ER cisternae being observed microscopically (Ramamohan et al., 2007; Vijayalakshmi et al., 2011; Brauer et al., 2020). Mitochondrial and lysosomal dysregulation have also been described, hinting at possible oxidative stress (Sharma et al., 2016). While apoptosis is another downstream feature of ALS-CSF exposure (Vijayalakshmi et al., 2011), it is again unclear whether this could be a direct consequence of the various ultrastructural observations.

In addition to cellular changes, intrathecal and intraventricular infusion of ALS-CSF in rat models were also found to result in motor impairment and other musculoskeletal features (Sankaranarayani et al., 2010, 2014; Sumitha et al., 2014; Shanmukha et al., 2018). These could possibly be attributed to altered neuronal activity in the motor cortex, in which increased neuronal excitability was observed (Sankaranarayani et al., 2010, 2014; Shanmukha et al., 2018). It has previously been shown that ALS-CSF exposure could lead to reduced expression levels of ion channels, including Na_v_1.6 and K_v_1.6 channels (Gunasekaran et al., 2009), although their exact association with motor changes is yet to be explored. The expression of miR-206, which is involved in muscle development, was also found to be altered by ALS-CSF infusion (Sumitha et al., 2014). A more recent study reported significant damage to the neuromuscular junction following intrathecal injection of ALS-CSF, along with mitochondrial and sarcoplasmic reticular defects (Shanmukha et al., 2018). The cellular changes produced by intrathecal administration of ALS-CSF have also been found to reflect the degree of motor impairment (Das et al., 2020). Overall, these findings suggest that mechanisms common to both ALS-CSF toxicity and ALS pathophysiology may exist beyond the cellular level.

## Neuroprotection From CSF Toxicity

Intriguingly, several neuroprotective factors have also emerged from CSF toxicity studies. VEGF, which has previously been implicated in ALS pathophysiology (Oosthuyse et al., 2001; Lambrechts et al., 2003; Azzouz et al., 2004; Storkebaum et al., 2005), has also been shown to counteract the neurotoxicity of ALS-CSF, reversing morphological changes and decreasing aggregation of phosphorylated neurofilaments (Kulshreshtha et al., 2011). In a later study, VEGF supplementation was further found to reduce caspase-3 levels and restore the expression of calbindin-D28K expression following ALS-CSF exposure, the latter possibly contributing to improved calcium buffering capacity (Vijayalakshmi et al., 2015). It is also worth pointing out that the expression of various growth factors, including brain-derived neurotrophic factor (BDNF), fibroblast growth factor 2 (FGF2) and insulin-like growth factor 1 (IGF1), is downregulated upon ALS-CSF infusion (Deepa et al., 2011). While the exact downstream consequences remain unclear, administration of BDNF, ciliary neurotrophic factor (CNTF) and platelet-derived growth factor (PDGF) has been shown to offer neuroprotection (Gunasekaran et al., 2009; Deepa et al., 2011; Chen et al., 2014; Shruthi et al., 2017).

## Is There a Consensus Regarding CSF Toxicity in ALS?

Despite the increasing recognition that ALS-CSF may promote neurodegeneration, with various studies having attempted to characterize its toxicity, synthesizing the current body of literature remains challenging (Box 1). Most prominently, substantial heterogeneity exists across studies with regard to the employed disease models and CSF exposure conditions (Ng Kee Kwong et al., 2020a). For instance, many studies have been performed using rat culture systems and NSC-34 cells, although results have recently been reproduced in human iPSC-derived and hESC-derived motor neurons (Sumitha et al., 2019; Brauer et al., 2020). We have also previously described considerable variation in how results are reported and interpreted across studies (Ng Kee Kwong et al., 2020a). Additionally, different patient and control populations are often recruited, the latter including subjects with wide-ranging conditions which could influence their CSF composition. However, we acknowledge the ethical implications of obtaining CSF from healthy controls. Along this line, it is also often not feasible to recruit larger patient populations in such studies.

**Box 1. Outstanding questions regarding CSF toxicity in ALS.**

• What is the nature of the circulating toxin responsible for CSF-induced neurodegeneration?
• What is the cellular origin of this circulating toxin?
• What is/are the mechanism/s by which it triggers neurodegeneration?
• Do astrocytes and microglia play a role in mediating CSF toxicity?
• To what extent does CSF composition reflect that of ISF?
• What is the contribution of CSF toxicity toward ALS pathophysiology?
• How accurately do the models employed to study CSF toxicity recapitulate human *in vivo* conditions?
• Could similar mechanisms explain CSF toxicity in sporadic and familial forms of ALS?

It is still unclear why conflicting findings have been obtained in early toxicity studies, both in those involving serum and CSF. Studies concomitantly investigating the toxicity of serum and CSF in ALS patients have, to our knowledge, not been conducted, and could potentially reveal additional insights into their association. Furthermore, no significant correlation has to date been observed by studies assessing the possible relationship between CSF toxicity and demographic and clinical variables, suggesting that CSF toxicity may not represent a possible biomarker for disease stratification (Tikka et al., 2002; Anneser et al., 2006; Barber et al., 2011; Yanez et al., 2011; Galan et al., 2017). Although the underlying reasons for this lack of association deserve further investigation, they could nonetheless help to confirm the nature of ALS as a highly heterogeneous disease. This would, notwithstanding methodological considerations, be consistent with recent evidence demonstrating that CSF cytotoxicity is not a feature of all ALS patients (Galan et al., 2017).

In spite of this, there are a number of advantages to considering the neurotoxicity of CSF in an ALS model. First, CSF toxicity, which has been observed across both sporadic and familial forms of ALS, could potentially form part of a common pathogenic pathway in ALS, thus helping to reconcile the two forms of ALS. Although its contribution in ALS pathophysiology has not yet been established, ALS-CSF toxicity appears to possess many common features, including an apparent predilection for motor neurons and an important cell non-autonomous component. As previously mentioned, the presence of a circulating toxin in CSF could also offer possible explanations for the spread of pathology in ALS (Smith et al., 2015). Future research may benefit from additional insights into the cellular origin of this circulating toxin, which has remained elusive to date. The possibility that toxicity may be a feature acquired within the CSF circulation, as has been proposed in the case of SOD1 misfolding (Tokuda et al., 2019), however, cannot be excluded.

**FIGURE 2:**
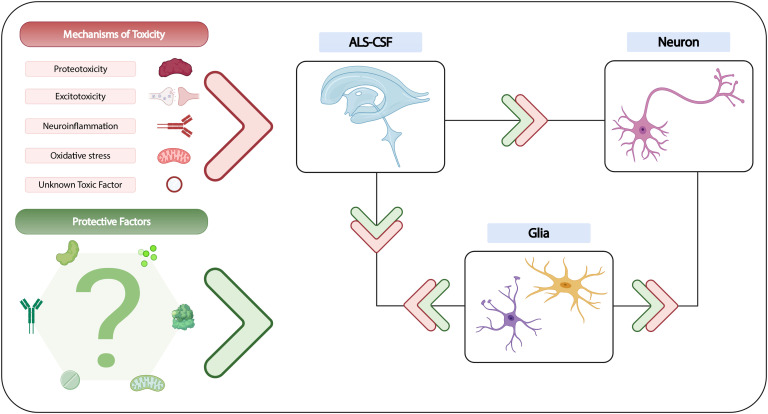
Summary of current understanding of CSF toxicity in ALS. While the exact cause of CSF-induced neurodegeneration remains to be established, various mechanisms of neurotoxicity have been evidenced, and could potentially be attributed to several candidate factors, of which some may not yet be known. The neuroprotective qualities of certain growth factors, glutamate receptor antagonists and antioxidants have also been demonstrated *in vitro*, although the contribution of protective factors in *in vivo* models is still unclear. Nevertheless, the existing literature suggests that a highly complex interplay may exist between ALS-CSF, glial cells and neurons, with the involvement of both toxic and protective factors that could directly or indirectly influence neuronal degeneration.

While drawing definite conclusions at this stage may not be possible, we believe, based on the numerous observations derived from past studies, that ALS-CSF contains both toxic and protective factors, and is able to promote neurodegeneration either directly or indirectly *via* a process mediated by both astrocytes and microglia (Figure 2). The neurodegenerative process may be driven by proteotoxicity or neuroinflammation, with possible contribution from other toxic components of CSF. Although evidence suggests that endogenous glutamate is unlikely to initiate the neurodegenerative process, glutamate excitotoxicity is likely to be an important step in the overall pathway. Other downstream consequences leading to the death of motor neurons may include Golgi fragmentation, mitochondrial dysfunction, oxidative stress and apoptosis.

Additional studies are nevertheless required to improve our understanding of CSF toxicity based on both its constitution and dynamics. Although proteomic and lipidomic studies have revealed altered levels of inflammatory markers as well as a different lipid profile in ALS-CSF (Barschke et al., 2017; Blasco et al., 2017; Hayashi et al., 2019), the literature surrounding proteomics in the study of CSF toxicity remains sparse (Varghese et al., 2013), despite its potential in revealing additional candidates for the toxicity of ALS-CSF. CSF biomarker studies have shown many ALS-associated proteins, including TDP-43 and neurofilaments, to be significantly elevated in ALS-CSF (Schreiber et al., 2018; Kasai et al., 2019). While it is unclear whether this may be due to increased production or reduced clearance, the contribution of CSF in their regulation may deserve investigation. Given the currently limited understanding of CSF dynamics, we also propose that investigating the interplay between the extracellular space and the CSF circulation, in which the glymphatic system has been shown to play an important role (Jessen et al., 2015; Plog and Nedergaard, 2018), may help to establish how accurately CSF constitution reflects that of the interstitium. Ependymal cells and astrocytes, which are in direct contact with the CSF, could potentially be vulnerable to its toxicity in ALS patients. Finally, we posit, based on recent evidence of CSF-mediated neurodegeneration in multiple sclerosis and Parkinson’s disease (Kong et al., 2015; Wentling et al., 2019), that CSF toxicity may well be a feature of other neurodegenerative diseases, thus adding to the growing list of common mechanisms that appear to underpin their pathophysiology.

## Conclusion

From initial findings of serum toxicity to current *in vivo* and *in vitro* evidence of CSF toxicity, striking similarities have been observed between CSF-mediated neurodegeneration and ALS pathophysiology. Although it would be difficult at this stage to argue for the role CSF toxicity as a driver of the disease process, we believe that the potential promise of CSF toxicity in capturing the broader pathophysiological picture of ALS may warrant its additional attention as an area of active research.

## Author Contributions

KN and AM conceptualized the work. KN wrote the first draft of the manuscript, supervised by AM. All authors provided critical input and revised subsequent drafts, before approving the final manuscript

## Conflict of Interest

The authors declare that the research was conducted in the absence of any commercial or financial relationships that could be construed as a potential conflict of interest.

## Abbreviations

AIDA: 1-aminoindan-1,5-dicarboxylic acid
ALS: amyotrophic lateral sclerosis
ALS-CSF: cerebrospinal fluid obtained from people with amyotrophic lateral sclerosis
AMPA: alpha-amino-3-hydroxy-5-methyl-4-isoxazole propionic acid
BDNF: brain-derived neurotrophic factor
CHIT-1: chitotriosidase-1
CNQX: 6-cyano-7-nitroquinoxaline-2,3-dione
CNS: central nervous system
CNTF: ciliary neurotrophic factor
CSF: cerebrospinal fluid
FGF2: fibroblast growth factor 2
FTD: frontotemporal dementia
FUS: fused in sarcoma
GLAST: glutamate aspartate transporter
GLT-1: glutamate transporter-1
hESC: human embryonic stem cell
HNE: 4-hydroxynonenal
IGF1: insulin-like growth factor 1
IgG: immunoglobulin G
IL-6: interleukin 6
IL-10: interleukin 10
iPSC: induced pluripotent stem cell
ISF: interstitial fluid
MAO-B: monoamine oxidase B
mGluR: metabotropic glutamate receptor
MND: motor neuron disease
NMDA: *N*-methyl -D-aspartate
NO: nitric oxide
NSC-34: mouse spinal cord-neuroblastoma hybrid cell line
NSE: neuron-specific enolase
PDGF: platelet-derived growth factor
ROS: reactive oxygen species
SOD1: superoxide dismutase 1
TDP-43: TAR DNA-binding protein 43
TLS: translocated in liposarcoma
TNF- α: tumor necrosis factor alpha
VEGF: vascular endothelial growth factor.
